# Drivers of antimicrobial resistance in layer poultry farming: Evidence from high prevalence of multidrug-resistant *Escherichia coli* and enterococci in Zambia

**DOI:** 10.14202/vetworld.2023.1803-1814

**Published:** 2023-09-14

**Authors:** Steward Mudenda, Flavien Nsoni Bumbangi, Kaunda Yamba, Musso Munyeme, Sydney Malama, Moses Mukosha, Mwendalubi Albert Hadunka, Victor Daka, Scott Kaba Matafwali, Godfrey Siluchali, Geoffrey Mainda, Mercy Mukuma, Bernard Mudenda Hang’ombe, John Bwalya Muma

**Affiliations:** 1 Department of Pharmacy, School of Health Sciences, University of Zambia, Lusaka, Zambia; 2 Department of Disease Control, School of Veterinary Medicine, University of Zambia, Lusaka, Zambia; 3 Department of Medicine and Clinical Sciences, School of Medicine, Eden University, Lusaka, Zambia; 4 Department of Biological Sciences, School of Natural Sciences, University of Zambia, Lusaka, Zambia; 5 Department of Pathology and Microbiology Laboratory, University Teaching Hospitals, Lusaka, Zambia; 6 Centre for Infectious Disease Research in Zambia, Lusaka, Zambia; 7 Department of Public Health, Michael Chilufya Sata School of Medicine, Copperbelt University, Ndola, Zambia; 8 Department of Clinical Research, Faculty of Infectious and Tropical Diseases, London School of Hygiene and Tropical Medicine, London, United Kingdom; 9 Department of Anatomy and Physiological Sciences, Institute of Basic and Biomedical Sciences, Levy Mwanawasa Medical University, Lusaka, Zambia; 10 Food and Agriculture Organization (FAO) of the United Nations, House No. 5 Chaholi, off Addis Ababa drive, Lusaka, Zambia; 11 Department of Food Science and Nutrition, School of Agricultural Sciences, University of Zambia, Lusaka, Zambia; 12 Department of Paraclinical Studies, School of Veterinary Medicine, University of Zambia, Lusaka, Zambia

**Keywords:** antimicrobial resistance, drivers, *Escherichia coli*, poultry, risk factors, Zambia

## Abstract

**Background and Aim:**

Inappropriate use of antimicrobials exacerbates antimicrobial resistance (AMR) in the poultry sector. Information on factors driving AMR in the layer poultry sector is scarce in Zambia. This study examined the drivers of AMR in the layer poultry sector in the Lusaka and Copperbelt Provinces of Zambia.

**Materials and Methods:**

This cross-sectional study employed a structured questionnaire in 77 layer poultry farms in the provinces of Lusaka and Copperbelt, Zambia, from September 2020 to April 2021. Data analysis was conducted using Stata version 16.1. Antimicrobial resistance was defined as the presence of multidrug resistance (MDR) isolates. Multivariable regression analysis was used to identify drivers of AMR.

**Results:**

In total, 365 samples were collected, from which 339 (92.9%) *Escherichia coli* and 308 (84.4%) *Enterococcus* spp. were isolated. Multidrug resistance was identified in 39% of the *E. coli* and 86% of the *Enterococcus* spp. The overall prevalence of AMR in layer poultry farms was 51.7% (95% confidence interval [CI]: 40.3%–63.5%). Large-scale farmers (Adjusted odds ratio [AOR] = 0.20, 95% CI: 0.04%–0.99%) than small-scale and farmers who were aware of AMR than those who were unaware (AOR = 0.26, 95% CI: 0.08%–0.86%) were less likely to experience AMR problems.

**Conclusion:**

This study found a high prevalence of AMR in layer poultry farming linked to the type of farm management practices and lack of AMR awareness. Evidence of high MDR in our study is of public health concern and requires urgent attention. Educational interventions must increase AMR awareness, especially among small- and medium-scale poultry farmers.

## Introduction

Antimicrobial resistance (AMR) is a public health problem that has been linked to the inappropriate use of antibiotics [1–4] and increased morbidity and mortality worldwide [5, 6]. Antimicrobial resistance has increased medical costs and negatively impacted global economy [7, 8]. Furthermore, the emergence of multidrug resistance (MDR) has made the treatment of infections challenging [9–11]. If MDR is not addressed, it is estimated to cause many deaths and lead to the next pandemic [12–17].

The development of AMR in poultry is complex and has been linked to many factors [18, 19]. In addition to bacterial resistance [20–22], overuse and misuse of antibiotics have worsened the AMR problem [23–27]. Most farmers use antibiotics to promote growth, prevent infections, and treat microbial infections in livestock and plants [28–33]. This may cause microorganisms to develop resistance to different classes of antimicrobials [29, 34]. The inappropriate use of antibiotics in poultry has contributed to increased AMR of *Escherichia coli* and enterococci [35–38]. These are commensal bacteria in the gastrointestinal tract of animals, but may become pathogenic once they develop AMR or acquire virulence factors [39–43].

Most antibiotics for humans and animals are prescribed empirically, without confirming the causative pathogen and its antimicrobial susceptibility [44–46]. In addition, most antibiotics are prescribed in lower doses and for a shorter duration, leading to persistent subpatent disease [45, 47, 48]. Conversely, some prescribers tend to prescribe antibiotics for a longer period, increasing the risk of AMR [49, 50]. This has been worsened by a lack of laboratory diagnostic facilities for pathogen detection and susceptibility testing, leading to wrong drug prescriptions [51]. Most of these practices have been exacerbated by easy access to antibiotics, usually without prescription [52, 53].

Reports have shown that a lack of awareness and knowledge of antimicrobial use (AMU) among veterinary drug dispensers has contributed to the development of antimicrobial-resistant infections in poultry [54, 55]. Veterinary drug vendors or agrovet dispensers often possess inadequate knowledge regarding poultry AMU and AMR, resulting in insufficient information provided to the farmers about antibiotic indications, dose, frequency, and duration [56]. In addition, veterinary drug vendors do not provide adequate information to farmers on the importance of biosecurity, withdrawal period, use of prescriptions, and veterinary expert consultations [53]. This may lead to the irrational use of antimicrobials in poultry and increase the risk of AMR. Consistently, the risks of AMR emergence and spread are high when poultry farmers have poor awareness and knowledge about AMU and AMR [57–59], leading to the misuse of antibiotics [60–63]. Furthermore, farmers with poor knowledge of AMR tend to be unaware of the withdrawal period of antibiotics, risking AMR development in poultry products and increasing the possibility of transmitting AMR pathogens to humans [64, 65].

The lack of standard treatment guidelines in the poultry sector has contributed to the development of AMR in some countries [66, 67]. Under these circumstances, there is no standardized treatment for diseases, and laboratory results do not inform the prescription of antimicrobials [66]. In humans, standard treatment guidelines are critical for promoting consistency, diagnosing correctly, and treating disease, thereby improving patient quality of care [68, 69]. Similarly, the development and implementation of, as well as adherence to, standard treatment guidelines in animal health are critical in promoting the rational use of antibiotics and reducing AMR [70–72].

Poor regulation of access to antibiotics is one of the major drivers of AMR [24, 73]. Unregulated antibiotic access causes individuals to have access to plenty, usually cheap, antibiotics without prescriptions, which promotes irrational use [24, 74]. Poor regulation of antibiotic use in poultry has negatively impacted human health [75]. In addition, poor disease and AMR surveillance systems have contributed to the emergence and spread of AMR [73, 76]. A lack of microbiological diagnostics, which are critical for monitoring the trends in AMR, has further worsened the problem [76, 77].

In Zambia, the excessive use of antibiotics in the poultry sector has contributed to the emergence of AMR [65, 78, 79]. Most Zambian poultry farmers have poor knowledge, negative attitudes, and poor practices toward AMU and AMR [65, 80]. The AMR situation in Zambia has been worsened by many factors [79]. Some of these factors include accessing antibiotics without prescription [65, 81], poor awareness of AMR [65, 80], inappropriate use of antibiotics [65, 78, 80, 81], and inappropriate prescribing of antibiotics [82–85].

Therefore, this study aimed to examine the drivers contributing to AMR development in layer poultry farming in the provinces of Lusaka and Copperbelt, Zambia.

## Materials and Methods

### Ethical approval and Informed consents

This study was approved by the ERES Converge ethics committee with a protocol ID # Ref No. 2019-Dec-004. We also obtained approval from the Zambia National Health Research Authority. All participants were informed about the purpose of the study, and they provided verbal and written consent to participate in the study. This study was conducted according to the guidelines of the Declaration of Helsinki.

### Study period and location

A cross-sectional study was conducted in layer poultry farms from September 2020 to April 2021 in the Copperbelt and Lusaka Provinces of Zambia. These two study sites produce the majority of Zambia’s poultry and poultry products [86] and have the highest number of households and populations [87, 88], which may translate into an increased need for poultry products. All poultry farmers involved in rearing layer hens in the production age who provided informed consent were included in the study. In addition, all layer chickens were to be healthy to minimize sampling of sick chickens that could have been on antimicrobial treatment. Chickens that were still in the withdrawal period after antimicrobial therapy were also excluded from this study.

### Sample size estimation

Sample size was estimated using Ausvet Epitools (https://epitools.ausvet.com.au/). The estimation was performed at 5% desired precision, 95% confidence level, and 50% estimated proportion, as described in a similar study [89]. Based on registers from the District Veterinary Offices and the Poultry Association of Zambia, the number of active layer poultry farms was estimated at 96 (56 in Lusaka and 40 in the Copperbelt). In total, 77 farms (45 from Lusaka and 32 from the Copperbelt) were included in this study based on consent from the farm owners.

### Data collection

#### Layer poultry farmers

We used a previously validated questionnaire to collect data from the farmers [90]. To allow for face and content validation, the questionnaire was reviewed by public health and epidemiological experts from the University of Zambia. This allowed for the prevalidation of the questionnaire for simplicity, relevance, accuracy, clarity, and logic. Next, we conducted a pilot study that included 12 farmers from Monze Township, Southern Province, Zambia, who were excluded from the final analysis of the main study findings. The pilot study helped assess the questionnaire’s consistency in collecting data. Cronbach’s α for the final questionnaire was 0.78, indicating acceptable internal consistency. The final questionnaire had two sections: Section A included questions on farm epidemiological data, such as type of farmer, gender of the owner, age of chickens, and type of floor; and section B included questions on antibiotic usage, use of prescriptions to access antibiotics, use of antibiotics to prevent infections, use of antibiotics to improve production, consulting veterinarians, administration of antibiotics, knowledge of withdrawal observation period, biosecurity practices at the farm, and awareness of AMR. Face-to-face interviews, which lasted for 20–30 min per participant, were conducted by three researchers. The participants were allowed to ask questions freely regarding poultry infections, antibiotics, and AMR. A brochure with information about AMR and its risk factors was given to the respondents after the interview.

### Poultry houses

All chickens that met the inclusion criteria were selected using a simple random method. Three chickens were randomly selected per 25 m^2^ of the poultry house. The cloacal region of chicken was swabbed to collect samples, which were then pre-enriched in 10 mL of buffered peptone water (BPW) (Oxoid, Basingstoke, UK). Samples collected in Lusaka were transported to the Public Health Laboratory at the School of Veterinary Medicine, University of Zambia, within 8 h. Samples collected in the Copperbelt province were placed in BPW immediately after collection, stored at the district veterinary offices at 2°C–8°C, and transported a day later to Lusaka for further processing. In total, 365 cloacal swab samples were collected and processed to isolate and identify *E. coli* and *Enterococcus* spp.

### Isolation and identification and confirmation of *E. coli* and enterococci

The isolation and identification of *E. coli* were performed using conventional methods and biochemical tests, as described by Mudenda *et al*. [91]. Presumptive *E. coli* isolates were confirmed using 16S rRNA gene sequencing [92]. Enterococci isolation and identification were performed using standard operating procedures, as described by Mudenda *et al*. [93], and confirmed using 16S rRNA gene sequencing [93].

### Antimicrobial susceptibility testing for *E. coli* and enterococci

Antimicrobial susceptibility testing was performed using the disk diffusion test [91, 94]. *Escherichia coli* and enterococci were exposed to a panel of 13 and 9 antibiotics of human and poultry importance in Zambia, respectively. Antibiotics were chosen based on the World Health Organization recommendations to test when conducting surveillance studies [95]. The zones of inhibition were categorized qualitatively as resistant (R), intermediate (I), and susceptible (S), and interpreted using the 2020 Clinical and Laboratory Standard Institute guidelines [96]. Resistance of isolates to ≥3 antibiotics from different classes was referred to as MDR [97].

### Statistical analysis

The collected data were entered in Microsoft Excel (Microsoft, Redmond, WA, USA), and statistical analyses were performed using Stata version 16.1 (Stata Corp., College Station, Texas, USA). The zones of inhibition were analyzed using WHONET 2020 (https://whonet.org/software.html) and reported as R, I, and S. Because the continuous variable (age of chickens) was not normally distributed (confirmed by using QQ-plot), it was summarized using the median and interquartile range (IQR). Categorical variables were reported as frequencies (%). The Wilcoxon-rank sum test, Fisher’s exact test, and Pearson’s Chi-square test were used where appropriate. The prevalence of AMR was calculated by dividing the number of AMR cases by the total number of respondents assessed and then multiplying by 100. Clopper-Pearson’s exact method was used to calculate 95% binomial confidence interval (CI) of the proportion of AMR cases. In this study, a farm was classified as having AMR if at least one isolate of *E. coli* or enterococci recovered from the farm had resistance to at least one antibiotic from three classes (positive AMR status = 1 and negative AMR status = 0). Univariate and multivariate binary logistic regression were used to assess AMR-related factors. Any variable with p < 0.2 from the univariate analysis was included to build the multivariate model. To avoid inflating the rate of type I error, the continuous variable (age of chickens) was not categorized in regression analysis. Investigator-led best model selection was used to build the final model. Forward and backward selection methods were used to obtain a parsimonious model. Differences in deviances were used to assess any possible terms to add to the final model. Assessment of interactions between significant variables in the final model showed no statistical significance. The Hosmer-Lemeshow goodness-of-fit statistic was used to further assess the goodness-of-fit of the final model. Two-sided p < 0.05 was considered statistically significant.

## Results

### Basic characteristics of layer poultry farms

Overall, 77 layer poultry farmers were enrolled in this study, of which 70 (90.9%) were males. Approximately one-half (39 [50.7%]) of the respondents were large-scale farmers, and 39 (50.7%) reported never using prescriptions when accessing antimicrobials. A larger proportion (66 [85.7%]) used antibiotics for poultry, 66 (85.7%) never consulted a veterinarian, 45 (58.4%) used antibiotics for infection prophylaxis, and 40 (52.0%) used antimicrobials to improve production. Nearly all (90.9%) who owned a farm were male, 75 (97.4%) had a concrete type of floor in the poultry house, and 70 (90.9%) had biosecurity measures in place. Furthermore, 61 (79.2%) reported using farm workers to administer antimicrobials, 48 (62.3%) knew about the withdrawal period, and 41 (53.3%) were aware of AMR. The overall median age of chickens at the time of assessment was 53 weeks (IQR: 38–68). In addition, there was evidence of an association between AMR and the person who administered antibiotics (p = 0.038) and awareness of AMR (p = 0.015) (Table-1).

**Table-1:** Basic characteristics of layer farms by the AMR status (n = 77).

Characteristic	Total population n (%)	Antimicrobial resistance		p-value

		No, n = 37 (%)	Yes, n = 40 (%)	
Type of farmer				
Small-scale	18 (23.4)	6 (16.2)	12 (30.0)	0.348
Medium-scale	20 (26.0)	11 (29.7)	9 (22.5)	
Large-scale	39 (50.7)	20 (54.1)	19 (47.5)	
Gender of owner				
Male	70 (90.9)	36 (97.3)	34 (85.0)	0.110
Female	7 (9.1)	1 (2.7)	6 (15.0)	
Age of chickens median (IQR)	53 (38–68)	54 (48–68)	51.5 (36–68.5)	0.482
Type of floor				
Concrete	75 (97.4)	36 (97.3)	39 (97.5)	1.000
Soil	2 (2.6)	1 (2.7)	1 (2.5)	
Antibiotic use				
Yes	66 (85.7)	34 (91.9)	32 (80.0)	0.136
No	11 (14.3)	3 (8.1)	8 (20.0)	
Use of prescription				
Yes	38 (49.4)	17 (46.0)	21 (52.5)	0.565
No	39 (50.7)	20 (54.1)	19 (47.5)	
Prevention of infection				
Yes	45 (58.4)	25 (67.6)	20 (50.0)	0.118
No	32 (41.6)	12 (32.4)	20 (50.0)	
Improving production				
Yes	37 (48.1)	17 (46.0)	20 (50.0)	0.722
No	40 (52.0)	20 (54.1)	20 (50.0)	
Consulting a veterinarian				
Yes	66 (85.7)	30 (81.1)	36 (90.0)	0.264
No	11 (14.3)	7 (18.9)	4 (10.0)	
Antibiotic administration				
Farm owner	16 (20.8)	4 (10.8)	12 (30.0)	**0.038**
Farm worker	61 (79.2)	33 (89.2)	28 (70.0)	
Knowledge of the observation period				
Yes	48 (62.3)	23 (62.2)	25 (62.5)	0.976
No	29 (37.7)	14 (37.8)	15 (37.5)	
Biosecurity practices				
Yes	70 (90.9)	32 (86.5)	38 (95.0)	0.251
No	7 (9.1)	5 (13.5)	2 (5.0)	
Aware of AMR				
Yes	36 (46.8)	12 (32.4)	24 (60.0)	**0.015**
No	41 (53.3)	25 (67.6)	16 (40.0)
Overall prevalence of AMR, % (95% CI)	51.9 (40.3–63.5)			

Fischer’s exact/Pearson Chi-square/Wilcoxon Rank sum test, IQR=Interquartile range, 95% CI=95% confidence interval, AMR=Antimicrobial resistance, bold values represent statistical significance at p < 0.05

### Antimicrobial susceptibility tests for *E. coli*

The findings demonstrated that *E. coli* isolates were highly resistant to tetracycline (54.6%), ampicillin (54.0%), and cefotaxime (30.4%) and highly susceptible to meropenem (94.7%), chloramphenicol (85.8%), and ceftazidime (85.3%) (Table-2).

**Table-2:** Antimicrobial resistance patterns of *Escherichia coli* isolates.

Antibiotic name	n (%) R	n (%) I	n (%) S	%R 95%CI
Amoxicillin/Clavulanic acid	25 (7.4)	32 (9.4)	282 (83.2)	13.1–21.3
Ampicillin	183 (54.0)	40 (11.8)	116 (34.2)	48.5–59.4
Cefotaxime	103 (30.4)	39 (11.5)	197 (58.1)	25.6–35.6
Ceftazidime	21 (6.2)	29 (8.6)	289 (85.3)	4.0–9.5
Cefepime	21 (6.2)	61 (18.0)	257 (75.8)	4.0–9.5
Chloramphenicol	30 (8.8)	18 (5.3)	291 (85.8)	6.1–12.5
Ciprofloxacin	86 (25.4)	80 (23.6)	173 (51.0)	20.9–30.4
Gentamicin	29 (8.6)	69 (20.4)	241 (71.1)	5.9–12.2
Meropenem	3 (0.9)	15 (4.4)	321 (94.7)	0.2–2.8
Nitrofurantoin	41 (12.1)	72 (21.2)	226 (66.7)	8.9–16.2
Tetracycline	184 (54.3)	52 (15.3)	103 (30.4)	49.1–59.9
Trimethoprim/Sulfamethoxazole	90 (26.5)	12 (3.5)	237 (69.9)	22.0–31.6
Nalidixic acid	82 (24.2)	58 (17.1)	199 (58.7)	19.8–29.2

R=Resistant, I=Intermediate, S=Susceptible, 95% CI=95% Confidence interval, n=number of isolates

### Antimicrobial susceptibility tests for enterococci

Enterococci isolates were highly resistant to tetracycline (80.5%), erythromycin (53.6%), and quinupristin-dalfopristin (53.2%), but highly susceptible to nitrofurantoin (77.6%) and chloramphenicol (71.1%) (Table-3).

**Table-3:** Antimicrobial resistance patterns of *Enterococcus* spp. (n = 308).

Antibiotic name	n (%) R	n (%) I	n (%) S	% R 95% CI
Ampicillin	113 (36.7)	-	195 (63.3)	31.3–42.4
Chloramphenicol	12 (3.9)	77 (25)	219 (71.1)	2.1–6.9
Ciprofloxacin	34 (11.0)	126 (40.9)	148 (48.1)	7.9–15.2
Erythromycin	165 (53.6)	107 (34.7)	36 (11.7)	47.8–59.2
Linezolid	93 (30.2)	51 (16.6)	164 (53.2)	25.2–35.7
Nitrofurantoin	20 (6.5)	49 (15.9)	239 (77.6)	4.1–10.0
Quinupristin-dalfopristin	164 (53.2)	68 (22.1)	76 (24.7)	47.5–58.9
Tetracycline	248 (80.5)	22 (7.1)	38 (12.3)	75.6–84.7
Vancomycin	101 (32.8)	71 (23.1)	136 (44.2)	27.6–38.4

R=Resistant, I=Intermediate, S=Susceptible, 95% CI=95% Confidence interval, n=number of isolates

The prevalence of AMR in *E. coli* isolates was 96.5% (95% CI: 93.73%–98.07%), of which 64.6% (95% CI: 59.22%–69.64%) were MDR. The prevalence of AMR in *Enterococcus* spp. was 99.4%, of which 86.0% (95% CI: 81.7%–89.5%) were MDR (Table-4).

**Table-4:** Prevalence, AMR, and MDR of *E. coli* (n = 339) and *Enterococcus* species (n = 308).

Characteristics	*Escherichia coli* n (%)	*Enterococcus* species n (%)
Prevalence	339 (92.9)	308 (84.4)
AMR	327 (96.5)	306 (99.4)
MDR	219 (64.6)	265 (86.0)

AMR=Antimicrobial resistance, MDR=Multidrug resistance

**Figure-1:**
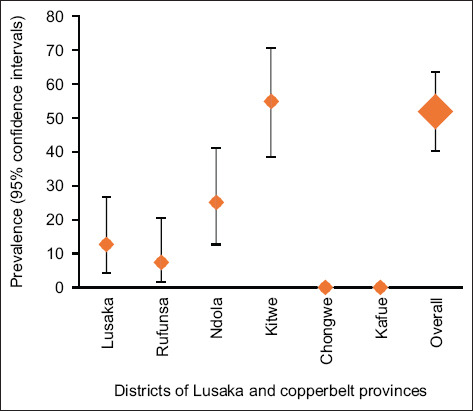
Prevalence of antimicrobial resistance problem at farm level among surveyed districts of Lusaka and Copperbelt provinces.

The prevalence of AMR at the farm level is shown in Figure-1. The overall prevalence of AMR was 51.9% (95% CI: 40.3%–63.5%), with the highest being in Kitwe district at 55% (95% CI: 38.5%–70.7%), followed by Ndola district at 25% (95% CI: 12.7%–41.2%), and Lusaka at 12.5% (95% CI: 4.2%–26.8%). Conversely, the lowest prevalence of AMR was in Rufunsa district at 7.5% (95% CI: 1.6%–20.4%).

Results from multivariable logistic regression analysis are shown in Table-5. Factors associated with AMR included the type of farmer and awareness of AMR. Compared to small-scale farmers, large-scale farmers were less likely to have AMR problems (Adjusted odds ratio [AOR] = 0.20, 95% CI: 0.04%–0.99%, p = 0.049). In addition, farmers who were aware of AMR were less likely to have AMR problems on their farms than those who were unaware (AOR = 0.26, 95% CI: 0.08%–0.86%, p = 0.027).

**Table-5:** Factors associated with AMR in layer poultry farms.

Characteristic	COR (95% CI)	p-value	AOR (95% CI)	p-value
Type of farmer				
Small-scale	Ref		Ref	
Medium-scale	0.41 (0.11–1.53)	0.184	0.31 (0.06–1.59)	0.161
Large-scale	0.48 (0.15–1.52)	0.210	0.20 (0.04–0.99)	**0.049**
Gender of owner				
Male	Ref			
Female	6.35 (0.73–55.54)	0.095		
Age of chickens	0.99 (0.98–1.02)	0.703		
Type of floor				
Concrete	Ref			
Soil	0.92 (0.06–15.31)	0.955		
Antibiotic use				
No	Ref		Ref	0.537
Yes	2.83 (0.69–11.63)	0.148	1.88 (0.25–14.05)	
Use of prescription				
No	Ref			
Yes	0.77 (0.31–1.88)	0.566		
Prevention of infection				
No	Ref			
Yes	2.08 (0.83–5.26)	0.120		
Improving production				
Yes	Ref			
No	0.85 (0.35–2.08)	0.722		
Consulting a veterinarian				
No	Ref			
Yes	0.48 (0.13–1.78)	0.271		
Antibiotic administration				
Farm owner	Ref		Ref	0.411
Farm worker	0.28 (0.08–0.98)	**0.046**	0.48 (0.09–2.74)	
Knowledge of the observation period				
No	Ref			
Yes	0.99 (0.39–2.48)	0.976		
Biosecurity practices				
No	Ref		Ref	0.223
Yes	0.34 (0.06–1.85)	0.211	0.27 (0.03–2.21)	
Aware of AMR				
No	Ref		Ref	**0.027**
Yes	0.32 (0.13–0.81)	**0.017**	0.26 (0.08–0.86)	

COR=Crude odds ratio, AOR=Adjusted odds ratio, 95% CI=95% confidence interval, AMR=Antimicrobial resistance, bold values represent statistical significance at p *<* 0.05, variables not appearing in the final multivariable model (blank spaces) were dropped from the final model

## Discussion

This study identified the drivers of AMR in layer poultry farms in Lusaka and Copperbelt provinces using *E. coli* and enterococci as indicator organisms. The study found that the prevalence of AMR in layer poultry farms was 51.9%. The prevalence of AMR for the isolated organism was 92.6% for *E. coli* and 84.4% for *Enterococcus* spp. The prevalence of MDR for *E. coli* was 39% and that of *Enterococcus* spp. was 86%. Large-scale poultry farmers were less likely to have AMR in their poultry than small-scale farmers, because commercial farmers employed professionals trained in managing an enterprise. In addition, farmers who were aware of AMR were less likely to have AMR in their poultries than those who were unaware.

In our study, the prevalence of MDR was high, translating into a serious AMR problem in layer poultry farms. This was evidenced by the high AMR and MDR rates of *E. coli* and enterococci. Consistently, other studies have reported high AMR rates of *E. coli* and enterococci [91, 93, 98–100]. The findings from our study and other studies may be partly due to the inappropriate use of antibiotics in the poultry sector [65]. In addition, these findings could be due to the natural phenomenon of AMR, where *E. coli* and enterococci develop natural resistance to antibiotics [101]. Therefore, the observed AMR levels in this study in both *E. coli* and enterococci were too high to be solely accounted for by the evolution of natural resistance.

This study found that large-scale layer poultry farmers were less likely to encounter AMR problems in their flocks. These findings are similar to those reported in China, where small- and medium-scale poultry farmers were more likely to misuse antibiotics than commercial farmers [102]. A study in Bangladesh reported that small-scale farmers were more likely to use antibiotics without consulting veterinary professionals, thereby increasing the risk of AMR in their farms [103]. Consequently, all small-scale poultry farmers were reported to have accessed antibiotics from their feed and chick sellers and used them to promote the growth of their chickens [103]. This practice was inappropriate because the poultry farmers missed out on expert information from pharmacy professionals and animal health personnel. Small-scale poultry farmers tend to misuse and overuse antibiotics due to a lack of antibiotic regulation, resources, and access to professional veterinary services [104]. Large-scale farmers are involved in rearing a larger number of layer chickens than small- and medium-scale farmers; hence, they usually vaccinate their layers to prevent infections [90]. Large-scale farmers are more likely to adhere to biosecurity guidelines than small- and medium-scale farmers, because they have to secure their high capital investment [90]. Adherence to biosecurity practices reduces the use of antibiotics in animal health [32, 105–107], and subsequently, reduces the risk of AMR development. In addition, large-scale farmers may employ knowledgeable employees who can follow the rules of biosecurity and vaccination. This could explain why medium- and large-scale farms have lower AMR rates than small-and medium-scale farms.

Our findings showed that layer poultry farmers who were aware of AMR were less likely to have AMR problems on their farms than those who were unaware. These findings agree with those of other studies that reported low awareness of AMR among poultry farmers [53, 59–61, 64]. A study in Bangladesh reported that most small-scale farmers had poor knowledge of AMR and farm management practices, resulting in increased risk of infection and overuse of antibiotics [108]. A study in Nigeria found that most poultry farmers who were unaware of AMR were inappropriately using antibiotics for growth promotion and disease prevention [109]. Similarly, a study in Nepal reported that most poultry farmers who were unaware of AMR were not compliant with the withdrawal period of antibiotics, used antimicrobials as growth promoters, and continuously used critically important antimicrobials, increasing the risk of AMR development [59]. Thus, policy enforcement is needed to monitor the use of antimicrobials in the poultry sector and improve outreach and educational activities in poultry farms [59]. In addition, AMR awareness must be promoted among farmers through sustainable mass media programs [110].

Our study revealed that most farmers used antibiotics that were accessed without prescriptions. Some farmers used antibiotics for prophylaxis and growth promotion. These findings are similar to those reported in other studies [53, 103, 109, 111, 112]. These practices by poultry farmers are potential contributing factors to the development of AMR. Therefore, there is a need for heightened antimicrobial stewardship and AMR surveillance programs in the poultry sector to increase awareness and promote the rational use of antibiotics [113–119]. Moreover, there is a need to promote behavioral change among poultry farmers regarding inappropriate AMU [119–122].

Our study highlights the potential drivers of AMR in poultry in Zambia. However, the results might have been better with a qualitative study because we collected data using a cross-sectional study. Consequently, we could not use focus group discussions due to the emphasis on adhering to the COVID-19 prevention measures. Despite these limitations, our findings concerning AMR and intervention measures in poultry are important for health authorities and decision-makers.

## Conclusion

This study found a high prevalence of AMR in layer poultry farming, especially among small-scale layer poultry farmers who were mostly unaware of AMR. The drivers of AMR identified in this study demonstrate the need to provide educational interventions to poultry farmers for disease prevention practices. Finally, there is a need to increase awareness of AMR and the contributing factors, especially among small- and medium-scale farmers. This can be done during outreach services in poultry farms and through activities, such as educational workshops and conferences, with the farmers.

## Authors’ Contributions

StM and JBM: Conceptualized the study. StM, KY, MAH, GM, and GS: Collected the data and conducted laboratory work. StM, MoM, and JBM: Performed data analysis. StM, FNB, KY, MuM, SyM, MoM, MAH, VD, SKM, GS, GM, MeM, BMH, and JBM interpreted the data. StM, KY, MuM, SyM, MoM, VD, SKM, FNB, GS, MeM, BMH, and JBM: Wrote the original draft preparation. StM, MuM, SyM, GM, MoM, KY, MAH, VD, SKM, FNB, GS, MeM, BMH, and JBM: Reviewed and edited the manuscript. StM, FNB, KY, MuM, SyM, MoM, MAH, VD, SKM, GM, MeM, BMH, and JBM revised the manuscript for important intellectual content. MuM, SyM, BMH, and JBM: Supervised the project. All authors have read, reviewed, and approved the final. manuscript.

## References

[1] Prestinaci F, Pezzotti P, Pantosti A (2015). Antimicrobial resistance:A global multifaceted phenomenon. Pathog. Glob. Health.

[2] Acharya K.P, Wilson R.T (2019). Antimicrobial resistance in Nepal. Front. Med.

[3] Mufwambi W, Stingl J, Masimirembwa C, Manasa J, Nhachi C, Stadler N, Mwila C, Kalungia A.C, Mukosha M, Mutiti C.S, Kamoto A, Kaonga P, Godman B, Munkombwe D (2021). Healthcare professionals'knowledge of pharmacogenetics and attitudes towards antimicrobial utilization in Zambia:Implications for a precision medicine approach to reducing antimicrobial resistance. Front. Pharmacol.

[4] Browne A.J, Chipeta M.G, Haines-Woodhouse G, Kumaran E.P.A, Hamadani B.H.K, Zaraa S, Henry N.J, Deshpande A, Reiner R.C, Day N.P.J, Lopez A.D, Dunachie S, Moore C.E, Stergachis A, Hay S.I, Dolecek C (2021). Global antibiotic consumption and usage in humans, 2000–18:A spatial modelling study. Lancet Planet. Health.

[5] Ikuta K.S, Swetschinski L.R, Robles Aguilar G, Sharara F, Mestrovic T, Gray A. P, Davis Weaver N, Wool E.E, Han C, Gershberg Hayoon A, Aali A, Abate S.M, Abbasi-Kangevari M, Abbasi-Kangevari Z, Abd-Elsalam S, Abebe G, Abedi A, Abhari A.P, Abidi H, Naghavi M (2022). Global mortality associated with 33 bacterial pathogens in 2019:A systematic analysis for the Global Burden of Disease Study 2019. Lancet.

[6] Murray C.J, Ikuta K.S, Sharara F, Swetschinski L, Robles Aguilar G, Gray A, Han C, Bisignano C, Rao P, Wool E, Johnson S.C, Browne A.J, Chipeta M.G, Fell F, Hackett S, Haines-Woodhouse G, Kashef Hamadani B.H, Kumaran E.A.P, McManigal B, Naghavi M (2022). Global burden of bacterial antimicrobial resistance in 2019:A systematic analysis. Lancet.

[7] Dadgostar P (2019). Antimicrobial resistance:Implications and costs. Infect. Drug Resist.

[8] Jonas O.B, Irwin A, Berthe F.C.J, Le Gall F.G, Marquez P (2017). Drug-Resistant Infections:A Threat to Our Economic Future. World Bank Report.

[9] Choudhuri A.H, Ahuja B, Biswas P.S, Uppal R (2019). Epidemiology of multidrug-resistant infections after inter ICU transfer in India. Indian J. Crit. Care Med.

[10] Roy K, Islam M.S, Paul A, Ievy S, Talukder M, Sobur M.A, Ballah F.M, Khan M.S.R, Rahman M.T (2022). Molecular detection and antibiotyping of multi-drug resistant *Enterococcus faecium* from healthy broiler chickens in Bangladesh. Vet. Med. Sci.

[11] Bertelloni F, Salvadori C, Moni A, Cerri D, Mani P, Ebani V.V (2015). Antimicrobial resistance in *Enterococcus* spp. Isolated from laying hens of backyard poultry flocks. Ann. Agric. Environ. Med.

[12] Sharma C, Rokana N, Chandra M, Singh B.P, Gulhane R.D, Gill J.P.S, Ray P, Puniya A.K, Panwar H (2018). Antimicrobial resistance:Its surveillance, impact, and alternative management strategies in dairy animals. Front. Vet. Sci.

[13] Matamoros-Recio A, Franco-Gonzalez J.F, Forgione R.E, Torres-Mozas A, Silipo A, Martín-Santamaría S (2021). Understanding the antibacterial resistance:Computational explorations in bacterial membranes. ACS Omega.

[14] Jindal A.K, Pandya K, Khan I.D (2015). Antimicrobial resistance:A public health challenge. Med. J. Armed Forces India.

[15] Laxminarayan R (2022). The overlooked pandemic of antimicrobial resistance. Lancet.

[16] Cameron A, Esiovwa R, Connolly J, Hursthouse A, Henriquez F (2022). Antimicrobial resistance as a global health threat:The need to learn lessons from the COVID-19 pandemic. Glob. Policy.

[17] Gautam A (2022). Antimicrobial resistance:The next probable pandemic. JNMA J. Nepal Med. Assoc.

[18] Laxminarayan R, Duse A, Wattal C, Zaidi A.K.M, Wertheim H.F.L, Sumpradit N, Vlieghe E, Hara G.L, Gould I.M, Goossens H, Greko C, So A.D, Bigdeli M, Tomson G, Woodhouse W, Ombaka E, Peralta A.Q, Qamar F.N, Mir F, Cars O (2013). Antibiotic resistance-the need for global solutions. Lancet Infect. Dis.

[19] Knight G.M, Costelloe C, Murray K.A, Robotham J.V, Atun R, Holmes A.H (2018). Addressing the unknowns of antimicrobial resistance:Quantifying and mapping the drivers of burden. Clin. Infect. Dis.

[20] Merker M, Tueffers L, Vallier M, Groth E.E, Sonnenkalb L, Unterweger D, Baines J.F, Niemann S, Schulenburg H (2020). Evolutionary approaches to combat antibiotic resistance:Opportunities and challenges for precision medicine. Front. Immunol.

[21] Wellington E.M.H, Boxall A.B.A, Cross P, Feil E.J, Gaze W.H, Hawkey P.M, Johnson-Rollings A.S, Jones D.L, Lee N.M, Otten W, Thomas C.M, Williams A.P (2013). The role of the natural environment in the emergence of antibiotic resistance in Gram-negative bacteria. Lancet Infect. Dis.

[22] Dcosta V.M, King C.E, Kalan L, Morar M, Sung W.W.L, Schwarz C, Froese D, Zazula G, Calmels F, Debruyne R, Golding G.B, Poinar H.N, Wright G.D (2011). Antibiotic resistance is ancient. Nature.

[23] Vaughn V.M, Hersh A.L, Spivak E.S (2022). Antibiotic overuse and stewardship at hospital discharge:The reducing overuse of antibiotics at discharge home framework. Clin. Infect. Dis.

[24] Saleem M, Deters B, de la Bastide A, Korzen M (2019). Antibiotics overuse and bacterial resistance. Ann. Microbiol. Res.

[25] Malik B, Bhattacharyya S (2019). Antibiotic drug-resistance as a complex system driven by socio-economic growth and antibiotic misuse. Sci. Rep.

[26] Martin M.J, Thottathil S.E, Newman T.B (2015). Antibiotics overuse in animal agriculture:A call to action for health care providers. Am. J. Public Health.

[27] Webb H.E, Angulo F.J, Granier S.A, Scott H.M, Loneragan G.H (2017). Illustrative examples of probable transfer of resistance determinants from food animals to humans:Streptothricins, glycopeptides, and colistin. F1000Res.

[28] Jambalang A.R, Buys E.M, Botha F.S (2017). Bacterial species from retailed poultry eggs in Tshwane, South Africa:Implication for consumers. S. Afr. J. Sci.

[29] Alonso C.A, Zarazaga M, Ben Sallem R, Jouini A, Ben Slama K, Torres C (2017). Antibiotic resistance in *Escherichia coli* in husbandry animals:The African perspective. Lett. Appl. Microbiol.

[30] Mehdi Y, Létourneau-Montminy M.P, Gaucher M.L, Chorfi Y, Suresh G, Rouissi T, Brar S.K, Côté C, Ramirez A.A, Godbout S (2018). Use of antibiotics in broiler production:Global impacts and alternatives. Anim. Nutr.

[31] Paintsil E.K, Ofori L.A, Akenten C.W, Fosu D, Ofori S, Lamshöft M, May J, Danso K.O, Krumkamp R, Dekker D (2021). Antimicrobial usage in commercial and domestic poultry farming in two communities in the Ashanti region of Ghana. Antibiotics (Basel).

[32] Selaledi L.A, Hassan Z.M, Manyelo T.G, Mabelebele M (2020). The current status of the alternative use to antibiotics in poultry production:An African perspective. Antibiotics (Basel).

[33] Guetiya Wadoum R.E, Zambou N.F, Anyangwe F.F, Njimou J.R, Coman M.M, Verdenelli M.C, Cecchini C, Silvi S, Orpianesi C, Cresci A, Colizzi V (2016). Abusive use of antibiotics in poultry farming in Cameroon and the public health implications. Br. Poult. Sci.

[34] Jiménez-Belenguer A, Doménech E, Villagrá A, Fenollar A, Ferrús M.A (2016). Antimicrobial resistance of *Escherichia coli* isolated in newly-hatched chickens and effect of amoxicillin treatment during their growth. Avian Pathol.

[35] Imam T, Gibson J.S, Foysal M, Das S.B, Gupta S.D, Fournié G, Hoque M.A, Henning J (2020). A cross-sectional study of antimicrobial usage on commercial broiler and layer chicken farms in Bangladesh. Front. Vet. Sci.

[36] Fertner M.E, Olsen R.H, Bisgaard M, Christensen H (2011). Transmission and genetic diversity of *Enterococcus faecalis* among layer chickens during hatch. Acta Vet. Scand.

[37] Ferdous M.R.A, Ahmed M.R, Khan S.H, Mukta M.A, Anika T.T, Hossain M.T, Islam M.Z, Rafiq K (2020). Effect of discriminate and indiscriminate use of oxytetracycline on residual status in broiler soft tissues. Vet. World.

[38] Kiiti R.W, Komba E.V, Msoffe P.L, Mshana S.E, Rweyemamu M, Matee M.I.N (2021). Antimicrobial resistance profiles of *Escherichia coli* isolated from broiler and layer chickens in arusha and Mwanza, Tanzania. Int. J. Microbiol.

[39] Islam M.S, Hossain M.J, Sobur M.A, Punom S.A, Rahman A.M.M.T, Rahman M.T (2023). A systematic review on the occurrence of antimicrobial-resistant *Escherichia coli* in poultry and poultry environments in Bangladesh between 2010 and 2021. Biomed. Res. Int.

[40] Ramos S, Silva V, de Lurdes Enes Dapkevicius M, Caniça M, Tejedor-Junco M.T, Igrejas G, Poeta P (2020). *Escherichia coli* as commensal and pathogenic bacteria among food-producing animals:Health implications of extended-spectrum β-lactamase (ESBL) production. Animals (Basel).

[41] Ramos S, Silva V, Maria de Lurdes Enes Dapkevicius M, Igrejas G, Poeta P (2020). Enterococci, from harmless bacteria to a pathogen. Microorganisms.

[42] Ribeiro J, Silva V, Monteiro A, Vieira-Pinto M, Igrejas G, Reis F.S, Barros L, Poeta P (2023). Antibiotic resistance among gastrointestinal bacteria in broilers:A review focused on *Enterococcus* spp. and *Escherichia coli*. Animals (Basel).

[43] Mak P.H.W, Rehman M.A, Kiarie E.G, Topp E, Diarra M.S (2022). Production systems and important antimicrobial resistant-pathogenic bacteria in poultry:A review. J. Anim. Sci. Biotechnol.

[44] Fleming-Dutra K.E, Hersh A.L, Shapiro D.J, Bartoces M, Enns E.A, File T.M, Finkelstein J.A, Gerber J.S, Hyun D.Y, Linder J.A, Lynfield R, Margolis D.J, May L.S, Merenstein D, Metlay J.P, Newland J.G, Piccirillo J.F, Roberts R.M, Sanchez G.V.,…, Hicks L.A (2016). Prevalence of inappropriate antibiotic prescriptions among us ambulatory care visits, 2010–2011. JAMA.

[45] Llor C, Bjerrum L (2014). Antimicrobial resistance:Risk associated with antibiotic overuse and initiatives to reduce the problem. Ther. Adv. Drug Saf.

[46] Murillo-Zamora E, Trujillo X, Huerta M, Mendoza-Cano O, Guzmán-Esquivel J, Guzmán-Solórzano J.A, Ochoa-Castro M.R, Ortega-Macías A.G, Zepeda-Anaya A.L, Oca V.R.M, de Ríos-Silva M, Lugo-Radillo A (2022). Empirical antibiotic prescribing in adult COVID-19 inpatients over two years in Mexico. Antibiotics (Basel).

[47] Sartelli M, Hardcastle T.C, Catena F, Chichom-Mefire A, Coccolini F, Dhingra S, Haque M, Hodonou A, Iskandar K, Labricciosa F.M, Marmorale C, Sall I, Pagani L (2020). Antibiotic use in low and middle-income countries and the challenges of antimicrobial resistance in surgery. Antibiotics (Basel).

[48] Iskandar K, Molinier L, Hallit S, Sartelli M, Hardcastle T.C, Haque M, Lugova H, Dhingra S, Sharma P, Islam S, Mohammed I, Naina Mohamed I, Hanna P.A, El Hajj S, Jamaluddin N.A.H, Salameh P, Roques C (2021). Surveillance of antimicrobial resistance in low- and middle-income countries:A scattered picture. Antimicrob. Resist. Infect. Control.

[49] Kistler C.E, Zimmerman S, Scales K, Ward K, Weber D, Reed D, McClester M, Sloane P.D (2017). The antibiotic prescribing pathway for presumed urinary tract infections in nursing home residents. J. Am. Geriatr. Soc.

[50] Akhtar A, Khan A.H, Zainal H, Ahmad Hassali M.A, Ali I, Ming L.C (2020). Physicians'perspective on prescribing patterns and knowledge on antimicrobial use and resistance in Penang, Malaysia:A qualitative study. Front. Public Health.

[51] van de Geijn G.J.M, Denker S, Meuleman-van Waning V, Koeleman H.G.M, Birnie E, Braunstahl G.J, Njo T.L (2016). Evaluation of new laboratory tests to discriminate bacterial from nonbacterial chronic obstructive pulmonary disease exacerbations. Int. J. Lab. Hematol.

[52] Kemp S.A, Pinchbeck G.L, Fèvre E.M, Williams N.J (2021). A cross-sectional survey of the knowledge, attitudes, and practices of antimicrobial users and providers in an area of high-density livestock-human population in Western Kenya. Front. Vet. Sci.

[53] Kariuki J.W, Jacobs J, Ngogang M.P, Howland O (2023). Antibiotic use by poultry farmers in Kiambu County, Kenya:Exploring practices and drivers of potential overuse. Antimicrob. Resist. Infect. Control.

[54] Wangmoi K, Dorji T, Pokhrel N, Dorji T, Dorji J, Tenzin T (2021). Knowledge, attitude, and practice on antibiotic use and antibiotic resistance among the veterinarians and para-veterinarians in Bhutan. PLoS One.

[55] Aworh M.K, Kwaga J.K.P, Okolocha E.C (2021). Assessing knowledge, attitude, and practices of veterinarians towards antimicrobial use and stewardship as drivers of inappropriate use in Abuja, Nigeria. One Health Outlook.

[56] Norris J.M, Zhuo A, Govendir M, Rowbotham S.J, Labbate M, Degeling C, Gilbert G.L, Dominey-Howes D, Ward M.P (2019). Factors influencing the behaviour and perceptions of Australian veterinarians towards antibiotic use and antimicrobial resistance. PLoS One.

[57] Hassan M.M, Kalam M.A, Alim M.A, Shano S, Nayem M.R.K, Badsha M.R, Al Mamun M.A, Hoque A, Tanzin A.Z, Nath C, Khanom H, Khan S.A, Islam M.M, Uddin M.B, Islam A (2021). Knowledge, attitude, and practices on antimicrobial use and antimicrobial resistance among commercial poultry farmers in Bangladesh. Antibiotics (Basel).

[58] Sindato C, Mboera L.E.G, Katale B.Z, Frumence G, Kimera S, Clark T.G, Legido-Quigley H, Mshana S.E, Rweyemamu M.M, Matee M (2020). Knowledge, attitudes and practices regarding antimicrobial use and resistance among communities of Ilala, Kilosa and Kibaha districts of Tanzania. Antimicrob. Resist. Infect. Control.

[59] Lambrou A.S, Innes G.K, O'Sullivan L, Luitel H, Bhattarai R.K, Basnet H.B, Heaney C.D (2021). Policy implications for awareness gaps in antimicrobial resistance (AMR) and antimicrobial use among commercial Nepalese poultry producers. Glob. Health Res. Policy.

[60] Mund M.D, Khan U.H, Tahir U, Mustafa B.E, Fayyaz A (2017). Antimicrobial drug residues in poultry products and implications on public health:A review. Int. J. Food Properties.

[61] Ndukui J.G, Gikunju J.K, Aboge G.O, Mbaria J.M (2021). Antimicrobial use in commercial poultry production systems in Kiambu County, Kenya:A cross-sectional survey on knowledge, attitudes and practices. Open J. Anim. Sci.

[62] Alhaji N.B, Haruna A.E, Muhammad B, Lawan M.K, Isola T.O (2018). Antimicrobials usage assessments in commercial poultry and local birds in North-central Nigeria:Associated pathways and factors for resistance emergence and spread. Prev. Vet. Med.

[63] Islam M.Z, Islam M.S, Kundu L.R, Ahmed A, Hsan K, Pardhan S, Driscoll R, Hossain M.S, Hossain M.M (2022). Knowledge, attitudes and practices regarding antimicrobial usage, spread and resistance emergence in commercial poultry farms of Rajshahi district in Bangladesh. PLoS One.

[64] Tasmim S.T, Hasan M.M, Talukder S, Mandal A.K, Parvin M.S, Ali M.Y, Ehsan M.A, Islam M.T (2023). Socio-demographic determinants of use and misuse of antibiotics in commercial poultry farms in Bangladesh. Int. J. Infect. Dis.

[65] Mudenda S, Malama S, Munyeme M, Hang'ombe B.M, Mainda G, Kapona O, Mukosha M, Yamba K, Bumbangi F.N, Mfune R.L, Daka V, Mwenya D, Mpundu P, Siluchali G, Muma J.B (2022). Awareness of antimicrobial resistance and associated factors among layer poultry farmers in Zambia:Implications for surveillance and antimicrobial Stewardship programs. Antibiotics (Basel).

[66] Gray P, Jenner R, Norris J, Page S, Browning G (2021). Antimicrobial prescribing guidelines for poultry. Aust. Vet. J.

[67] Nhung N.T, Chansiripornchai N, Carrique-Mas J.J (2017). Antimicrobial resistance in bacterial poultry pathogens:A review. Front. Vet. Sci.

[68] Kruger M (2013). The importance of standard treatment guidelines in paediatric practice in Africa. Public Health Action.

[69] Wiedenmayer K, Ombaka E, Kabudi B, Canavan R, Rajkumar S, Chilunda F, Sungi S, Stoermer M (2021). Adherence to standard treatment guidelines among prescribers in primary healthcare facilities in the Dodoma region of Tanzania. BMC Health Serv. Res.

[70] Aidara-Kane A, Angulo F.J, Conly J, Minato Y, Silbergeld E.K, McEwen S.A, Collignon P.J, Balkhy H, Collignon P, Friedman C, Hollis A, Kariuki S, Kwak H.S, McEwen S, Moulin G, Ngandjio A, Rollin B, Rossi F, Wallinga D (2018). World Health Organization (WHO) guidelines on use of medically important antimicrobials in food-producing animals. Antimicrob. Resist. Infect. Control.

[71] Tebug S.F, Mouiche M.M.M, Abia W.A, Teno G, Tiambo C.K, Moffo F, Awah-Ndukum J (2021). Antimicrobial use and practices by animal health professionals in 20 sub-Saharan African countries. Prev. Vet. Med.

[72] Pugliese M, Voslarova E, Biondi V, Passantino A (2019). Clinical practice guidelines:An opinion of the legal implication to veterinary medicine. Animals (Basel).

[73] Gulumbe B.H, Haruna U.A, Almazan J, Ibrahim I.H, Faggo A.A, Bazata A.Y (2022). Combating the menace of antimicrobial resistance in Africa:A review on stewardship, surveillance and diagnostic strategies. Biol. Proced. Online.

[74] Michael C.A, Dominey-Howes D, Labbate M (2014). The antimicrobial resistance crisis:Causes, consequences, and management. Front. Public Health.

[75] Glasgow L, Forde M, Brow D, Mahoney C, Fletcher S, Rodrigo S (2019). Antibiotic use in poultry production in Grenada. Vet. Med. Int.

[76] Matee M, Mshana S.E, Mtebe M, Komba E.V, Moremi N, Lutamwa J, Kapona O, Sekamatte M, Mboera L.E.G (2023). Mapping and gap analysis on antimicrobial resistance surveillance systems in Kenya, Tanzania, Uganda and Zambia. Bull. Natl. Res. Cent.

[77] Ferguson J.K, Joseph J, Kangapu S, Zoleveke H, Townell N, Duke T, Manning L, Lavu E (2020). Quality microbiological diagnostics and antimicrobial susceptibility testing, an essential component of antimicrobial resistance surveillance and control efforts in Pacific island nations. Western Pac. Surveill. Response J.

[78] Chishimba K, Hang'ombe B.M, Muzandu K, Mshana S.E, Matee M.I, Nakajima C, Suzuki Y (2016). Detection of extended-spectrum beta-lactamase-producing *Escherichia coli* in market-ready chickens in Zambia. Int. J. Microbiol.

[79] Republic of Zambia NAP on AMR (2017). Multi-sectoral National Action Plan on Antimicrobial Resistance. Government of the Republic of Zambia.

[80] Chilawa S, Mudenda S, Daka V, Chileshe M, Matafwali S, Chabalenge B, Mpundu P, Mufwambi W, Mohamed S, Mfune R.L (2023). Knowledge, attitudes, and practices of poultry farmers on antimicrobial use and resistance in Kitwe, Zambia:Implications on antimicrobial Stewardship. Open J. Anim. Sci.

[81] Kalungia A.C, Burger J, Godman B, de Oliveira Costa J, Simuwelu C (2016). Non-prescription sale and dispensing of antibiotics in community pharmacies in Zambia. Expert Rev. Anti. Infect. Ther.

[82] Kalonga J, Hangoma J, Banda M, Munkombwe D, Mudenda S (2020). Antibiotic prescribing patterns in paediatric patients at levy Mwanawasa University Teaching Hospital in Lusaka, Zambia. Int. J. Pharm. Pharmacol.

[83] Kalungia A.C, Mukosha M, Mwila C, Banda D, Mwale M, Kagulura S, Ogunleye O.O, Meyer J.C, Godman B (2022). Antibiotic use and Stewardship indicators in the first- and second-level hospitals in Zambia:Findings and implications for the future. Antibiotics (Basel).

[84] Mudenda S, Chomba M, Chabalenge B, Hikaambo C.N, Banda M, Daka V, Zulu A, Mukesela A, Kasonde M, Lukonde P, Chikatula E, Matowe L, Mutati R.K, Muungo T.L, Mudenda T, Mohamed S, Matafwali S (2022). Antibiotic prescribing patterns in adult patients according to the WHO AWaRe classification:A multi-facility cross-sectional study in primary healthcare hospitals in Lusaka, Zambia. Pharmacol. Pharm.

[85] Mudenda S, Nsofu E, Chisha P, Daka V, Chabalenge B, Mufwambi W, Kainga H, Kanaan M.H.G, Mfune R.L, Mwaba F, Zulu M, Tembo R, Mwasinga W, Chishimba K, Mwikuma G, Monde N, Samutela M, Chiyangi H.K, Mohamed S, Matafwali S.K (2023). Prescribing patterns of antibiotics according to the WHO AWaRe classification during the COVID-19 pandemic at a teaching hospital in Lusaka, Zambia:Implications for strengthening of antimicrobial stewardship programmes. Pharmacoepidemiology.

[86] Krishnan S.B, Peterburs T (2017). Zambia Jobs in Value Chains:Opportunities in Agribusiness.

[87] ZamStats (2022). Population Size by Province, Zambia 2010 and 2022. Zambia Statistics Agency.

[88] ZamStats (2022). Total Number of Households by Province, Zambia 2022. Zambia Statistics Agency.

[89] Odoch T, Wasteson Y, L'Abée-Lund T, Muwonge A, Kankya C, Nyakarahuka L, Tegule S, Skjerve E (2017). Prevalence, antimicrobial susceptibility and risk factors associated with non-typhoidal *Salmonella* on Ugandan layer hen farms. BMC Vet. Res.

[90] Nkansa M, Agbekpornu H, Kikimoto B.B, Chandler C.I (2020). Antibiotic use among Poultry Farmers in the Dormaa Municipality, Ghana. Report for Fleming Fund Fellowship Programme.

[91] Mudenda S, Malama S, Munyeme M, Matafwali S.K, Kapila P, Katemangwe P, Mainda G, Mukubesa A.N, Hadunka M.A, Muma J.B (2023). Antimicrobial resistance profiles of *Escherichia coli* isolated from laying hens in Zambia:Implications and significance on one health. JAC Antimicrob. Resist.

[92] Muligisa-Muonga E, Mainda G, Mukuma M, Kwenda G, Hang'ombe B, Flavien B.N, Phiri N, Mwansa M, Munyeme M, Muma J.B (2021). Antimicrobial resistance of *Escherichia coli* and *Salmonella* isolated from retail broiler chicken carcasses in Zambia. J. Epidemiol. Res.

[93] Mudenda S, Matafwali S.K, Malama S, Munyeme M, Yamba K, Katemangwe P, Siluchali G, Mainda G, Mukuma M, Bumbangi F.N, Mirisho R, Muma J.B (2022). Prevalence and antimicrobial resistance patterns of Enterococcus species isolated from laying hens in Lusaka and Copperbelt provinces of Zambia:A call for AMR surveillance in the poultry sector. JAC Antimicrob. Resist.

[94] Bauer A.W, Kirby W.M, Sherris J.C, Turck M (1966). Antibiotic susceptibility testing by a standardized single disk method. Am. J. Clin. Pathol.

[95] McKenzie J.S, Morris R.S, Midwinter A, Burgess S, Amia W.C, Lopes H, Tuntasuvan D, Gordon N.C, Moyen N, Lesilie T (2019). A Protocol for Active AMR Surveillance in Poultry. Towards a One Health AMR Surveillance System :Protocol for Active AMR Surveillance in Commercial Broiler and Layer Chicken Populations for the Fleming Fund Grants Programme. Version 2 (Issue 12).

[96] Clinical and Laboratory Standards Institute (2020). Performance Standards for Antimicrobial Susceptibility Testing.

[97] Magiorakos A.P, Srinivasan A, Carey R.B, Carmeli Y, Falagas M.E, Giske C.G, Harbarth S, Hindler J.F, Kahlmeter G, Olsson-Liljequist B, Paterson D.L, Rice L.B, Stelling J, Struelens M.J, Vatopoulos A, Weber J.T, Monnet D.L (2012). Multidrug-resistant, extensively drug-resistant and pandrug-resistant bacteria:An international expert proposal for interim standard definitions for acquired resistance. Clin. Microbiol. Infect.

[98] Rafiq K, Islam M.R, Siddiky N.A, Samad M.A, Chowdhury S, Hossain K.M.M, Rume F.I, Hossain M.K, Mahbub-E-Elahi A, Ali M.Z, Rahman M, Amin M.R, Masuduzzaman M, Ahmed S, Ara Rumi N, Hossain M.T (2022). Antimicrobial resistance profile of common foodborne pathogens recovered from Livestock and poultry in Bangladesh. Antibiotics (Basel).

[99] Mahmud Z.H, Kabir M.H, Ali S, Moniruzzaman M, Imran K.M, Nafiz T.N, Islam M.S, Hussain A, Hakim S.A.I, Worth M, Ahmed D, Johnston D, Ahmed N (2020). Extended-spectrum beta-lactamase-producing *Escherichia coli* in drinking water samples from a forcibly displaced, densely populated community setting in Bangladesh. Front. Public Health.

[100] Hedman H.D, Vasco K.A, Zhang L (2020). A review of antimicrobial resistance in poultry farming within low-resource settings. Animals (Basel).

[101] Urban-Chmiel R, Marek A, Stępień-Pyśniak D, Wieczorek K, Dec M, Nowaczek A, Osek J (2022). Antibiotic resistance in bacteria--a review. Antibiotics (Basel).

[102] Xu J, Sangthong R, McNeil E, Tang R, Chongsuvivatwong V (2020). Antibiotic use in chicken farms in northwestern China. Antimicrob. Resist. Infect. Control.

[103] Al Masud A, Rousham E.K, Islam M.A, Alam M.U, Rahman M, Al Mamun A, Sarker S, Asaduzzaman M, Unicomb L (2020). Drivers of antibiotic use in poultry production in Bangladesh:Dependencies and dynamics of a patron-client relationship. Front. Vet. Sci.

[104] Liu B, Wang W, Deng Z, Ma C, Wang N, Fu C, Lambert H, Yan F (2023). Antibiotic governance and use on commercial and smallholder farms in eastern China. Front. Vet. Sci.

[105] Huber N, Andraud M, Sassu E.L, Prigge C, Zoche-Golob V, Käsbohrer A, D'Angelantonio D, Viltrop A, Żmudzki J, Jones H, Smith R.P, Tobias T, Burow E (2022). What is a biosecurity measure?A definition proposal for animal production and linked processing operations. One Health.

[106] Renault V, Humblet M.F, Saegerman C (2022). Biosecurity concept:Origins, evolution and perspectives. Animals (Basel).

[107] Scott A.B, Singh M, Groves P, Hernandez-Jover M, Barnes B, Glass K, Moloney B, Black A, Toribio J.A (2018). Biosecurity practices on Australian commercial layer and meat chicken farms:Performance and perceptions of farmers. PLoS One.

[108] Ferdous J, Sachi S, Al Noman Z, Hussani S.M.A.K, Sarker Y.A, Sikder M.H (2019). Assessing farmers'perspective on antibiotic usage and management practices in small-scale layer farms of Mymensingh district, Bangladesh. Vet. World.

[109] Chah J.M, Nwankwo S.C, Uddin I.O, Chah K.F (2022). Knowledge and practices regarding antibiotic use among small-scale poultry farmers in Enugu State, Nigeria. Heliyon.

[110] Harbarth S, Balkhy H.H, Goossens H, Jarlier V, Kluytmans J, Laxminarayan R, Saam M, Van Belkum A, Pittet D (2015). Antimicrobial resistance:One world, one fight!. Antimicrob. Resist. Infect. Control.

[111] Bamidele O, Amole T.A, Oyewale O.A, Bamidele O.O, Yakubu A, Ogundu U.E, Ajayi F.O, Hassan W.A (2022). Antimicrobial usage in smallholder poultry production in Nigeria. Vet. Med. Int.

[112] Chowdhury S, Fournié G, Blake D, Henning J, Conway P, Hoque M.A, Ghosh S, Parveen S, Biswas P.K, Akhtar Z, Islam K, Islam M.A, Rashid M.M, Pelligand L, Khan Z.H, Rahman M, Tomley F, Debnath N, Chowdhury F (2022). Antibiotic usage practices and its drivers in commercial chicken production in Bangladesh. PLoS One.

[113] Donado-Godoy P, Castellanos R, León M, Arevalo A, Clavijo V, Bernal J, León D, Tafur M.A, Byrne B.A, Smith W.A, Perez-Gutierrez E (2015). The establishment of the Colombian integrated program for antimicrobial resistance surveillance (COIPARS):A pilot project on poultry farms, slaughterhouses and retail market. Zoonoses Public Health.

[114] Caudell M.A, Kiambi S, Afakye K, Koka E, Kabali E, Kimani T, Dorado-Garcia A (2022). Social-technical interventions to reduce antimicrobial resistance in agriculture:Evidence from poultry Farmer Field Schools in Ghana and Kenya. JAC Antimicrob. Resist.

[115] Dyar O.J, Huttner B, Schouten J, Pulcini C (2017). What is antimicrobial stewardship?. Clin. Microbiol. Infect.

[116] Siachalinga L, Mufwambi W, Lee L.H (2022). Impact of antimicrobial stewardship interventions to improve antibiotic prescribing for hospital inpatients in Africa:A systematic review and meta-analysis. J. Hosp. Infect.

[117] Godman B, Egwuenu A, Haque M, Malande O.O, Schellack N, Kumar S, Saleem Z, Sneddon J, Hoxha I, Islam S, Mwita J, Do Nascimento R.C.R.M, Godói I.P.D, Niba L.L, Amu A.A, Acolatse J, Incoom R, Sefah I.A, Opanga S, Seaton R.A (2021). Strategies to improve antimicrobial utilization with a special focus on developing countries. Life.

[118] Mudenda S, Daka V, Matafwali S.K (2023). World Health Organization AWaRe framework for antibiotic stewardship:Where are we now and where do we need to go?An expert viewpoint. Antimicrob. Steward. Healthc. Epidemiol.

[119] Mudenda S, Chabalenge B, Daka V, Mfune R. L, Salachi K. I, Mohamed S, Mufwambi W, Kasanga M, Matafwali S. K (2023). Global strategies to combat antimicrobial resistance:A one health perspective. Pharmacol. Pharm.

[120] McKernan C, Benson T, Farrell S, Dean M (2021). Antimicrobial use in agriculture:Critical review of the factors influencing behaviour. JAC Antimicrob. Resist.

[121] Parveen S, Garzon-Orjuela N, Amin D, McHugh P, Vellinga A (2022). Public health interventions to improve antimicrobial resistance awareness and behavioural change associated with antimicrobial use:A systematic review exploring the use of social media. Antibiotics (Basel).

[122] Coyne L, Patrick I, Arief R, Benigno C, Kalpravidh W, McGrane J, Schoonman L, Sukarno A.H, Rushton J (2020). The costs, benefits and human behaviours for antimicrobial use in small commercial broiler chicken systems in Indonesia. Antibiotics (Basel).

